# Role of *IL-4* Gene Polymorphisms in HBV-Related Hepatocellular Carcinoma in a Chinese Population

**DOI:** 10.1371/journal.pone.0110061

**Published:** 2014-10-08

**Authors:** Yu Lu, Zhitong Wu, Qiliu Peng, Liping Ma, Xiaolian Zhang, Jiangyang Zhao, Xue Qin, Shan Li

**Affiliations:** 1 Department of Clinical Laboratory, First Affiliated Hospital of Guangxi Medical University, Nanning, Guangxi, China; 2 Department of Clinical Laboratory, Guigang People’s Hospital, Guigang, Guangxi, China; University of North Carolina School of Medicine, United States of America

## Abstract

**Background:**

Interleukin-4 (IL-4) is best known as an important mediator and modulator of immune and inflammatory responses. Hepatocellular carcinoma (HCC) is a typical inflammation-related cancer, and genetic variations in the *IL-4* gene may be associated with the risk of hepatitis B virus (HBV)-related HCC. However, few studies have been conducted on their association.

**Objectives:**

To clarify the effects of *IL-4* gene polymorphisms on the risk of HBV-related HCC, two common variants, −590C/T (rs2243250) and −33C/T (rs2070874), and their relationship with HBV-related disease risk were investigated in a Chinese population.

**Methods:**

*IL-4* −590C/T and −33C/T polymorphisms were examined in 154 patients with HBV-related HCC, 62 patients with HBV-induced liver cirrhosis (LC), 129 patients with chronic hepatitis B (CHB), and 94 healthy controls, using the polymerase chain reaction-restriction fragment length polymorphism method and DNA sequencing.

**Results:**

Overall, no significant differences were observed regarding the *IL-4* −590C/T and −33C/T polymorphism genotypes, alleles, or haplotypes between the patient groups and the healthy controls. However, the CC genotypes of *IL-4* −590C/T and −33C/T polymorphisms were observed to be significantly associated with CHB in subgroup analysis in males [CC versus TT (OR: 4.193, 95% CI: 1.094–16.071, *P* = 0.037; and OR: 3.438, 95% CI: 1.032–11.458, *P* = 0.044) and CC versus TT+CT (OR: 4.09, 95% CI: 1.08–15.49, *P* = 0.038; and OR: 3.43, 95% CI: 1.04–11.28, *P* = 0.042)].

**Conclusions:**

These findings suggest that genetic variants in *IL-4* −590C/T and −33C/T polymorphisms may be a risk factor for CHB in Chinese males but not for HBV-related LC or HCC.

## Introduction

Hepatitis B virus (HBV) infection is one of the leading causes of liver disease, with more than 2 billion people infected worldwide [1], [2]. Among them, approximately 350 million individuals persistently infected with HBV develop chronic hepatitis, which results in liver cirrhosis (LC) in one third of all cases, more than three quarters of which progress to hepatocellular carcinoma (HCC) [3]. It is widely accepted that chronic hepatitis B (CHB), LC, and HCC are progressive stages of chronic HBV infection [4]. Nevertheless, the mechanisms underlying such progression are not yet well understood, although immune system-mediated chronic inflammation is believed to play a pivotal role in HCC development. HBV clearance depends on an effective host immune response [5] and, thus, ineffective host immunity may create a hepatocarcinogenic microenvironment in the presence of HBV infection [6]–[8].

Interleukin-4 (IL-4), a multifunctional pleiotropic cytokine mainly produced by activated T helper 2 (Th2) cells, plays important roles as a mediator and modulator of immune and inflammatory responses [9]; it is not only involved in humoral and cell-mediated immunity, but also an essential regulator in the immune response of B cells, T cells, and macrophages to fight against infections and malignant cells [10]; Th2 diseases are, in their majority, driven by IL-4 [11]. In addition, IL-4 and IL-13 are the archetypal inducers of M2 [12]- an alternatively activated macrophage [13] that has been proved to promote HCC development [14]. Any alterations that influence the expression and function of IL-4 can lead to weakened immune responses, and thus increase the susceptibility to infections and inflammation-related diseases [15]. Due to their highly polymorphic nature, genetic mutations in this gene might be the major contributor.

The human *IL-4* gene is located on chromosome 5q31 and consists of 25 kbps [16]. So far, more than 50 allelic variant polymorphisms have been found (http://www.ncbi.nlm.nih.gov/SNP/), including −590C/T (rs2243250), −33C/T (rs2070874), +3437C/G (rs2227282), and 2979G/T (rs2227284) [17]. The impact of *IL-4* gene polymorphisms in the pathogenesis of HBV infection has been investigated, and numerous studies have suggested that *IL-4* variants play pivotal roles in hepatitis B vaccine response and HBV infection risk [18]–[21]. However, to date, few studies have been conducted to investigate the role of these polymorphisms in HCC development [22]. In the present study, the two most common promoter polymorphisms of *IL-4*, −590C/T (rs2243250) and −33C/T (rs2070874), were evaluated in four Chinese patient groups – patients with CHB, LC, and HBV-related HCC, and a healthy control group – to determine whether these *IL-4* gene polymorphisms contribute to the susceptibility of HBV-related HCC.

## Materials and Methods

### Study population

A hospital-based series of 345 unrelated patients were recruited from the First Affiliated Hospital of Guangxi Medical University (Guangxi, China) between June and November 2013, including 154 patients with HBV-related HCC, 62 patients with HBV-induced LC, and 129 patients with CHB. To confirm that all patients had a chronic HBV infection for a period of at least 6 months, hepatitis B surface antigen (HBsAg), hepatitis B virus core antibody, and hepatitis Be antigen or hepatitis Be antibody were confirmed as seropositive. CHB was further diagnosed with serum HBV-DNA levels >1,000 copies/mL, as well as elevated alanine aminotransferase or aspartate aminotransferase (>2 times the upper limit of normal) [23]. LC was diagnosed by histologic analysis of liver biopsy specimens or typical morphologic findings from computed tomography or ultrasonography, together with laboratory features. For the HCC group, the patients enrolled fulfilled the following criteria: i) positive cytologic or pathologic findings or ii) elevated α-fetoprotein levels (≥400 ng/mL) combined with a positive image on computed tomography or ultrasonography; iii) were newly diagnosed HCC patients without a prior medical history of HCC; and iv) not diagnosed with other cancers [24]. For comparison, 170 HBV free healthy controls were randomly selected from the Health Examination Center of the First Affiliated Hospital of Guangxi Medical University during the same period. The selection criteria for controls were absence of any malignancy or other serious illness, as well as other inflammatory status such as autoimmune hepatitis which may generate possible bais. All subjects were Chinese from the Guangxi District. Written informed consent was obtained from each participant, and the study was approved by the ethics committee of the First Affiliated Hospital of Guangxi Medical University, Nanning, Guangxi, China.

### DNA extraction and single nucleotide polymorphism (SNP) genotyping

**Figure 1:**
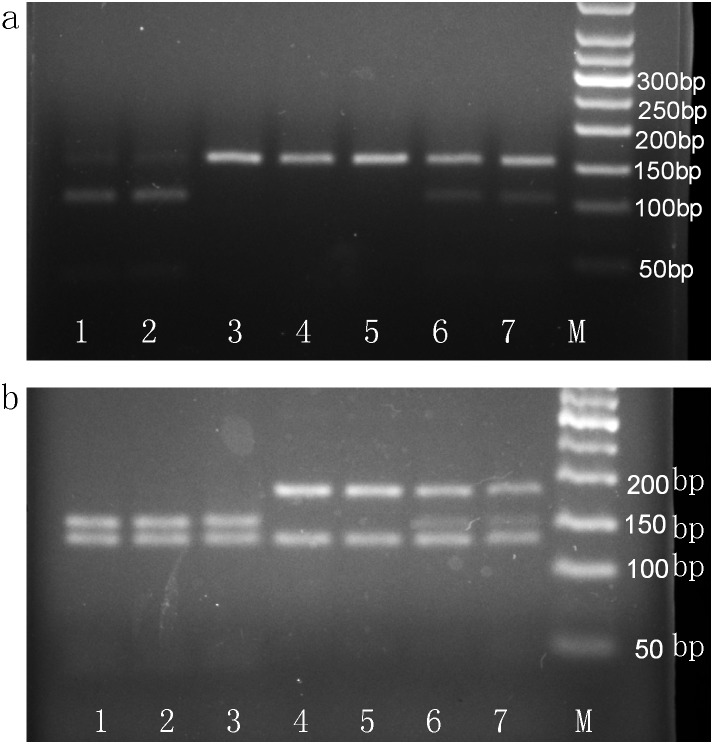
PCR-RFLP assay for analyzing the IL-4−589C/T and −33C/T polymorphisms of the IL-4 gene. PCR product was digested by restriction enzyme and visualized on a 2.5% agarose gel. a −589C/T polymorphism. Lanes 1 and 2 show CC genotype; lanes 3, 4 and 5 show TT genotype; lanes 6 and 7 show CT genotype. b −33C/T polymorphism. Lanes 1, 2 and 3 show TT genotype; lanes 4 and 5 show CC genotype; lanes 6 and 7 show CT genotype. M, marker.

**Figure 2:**
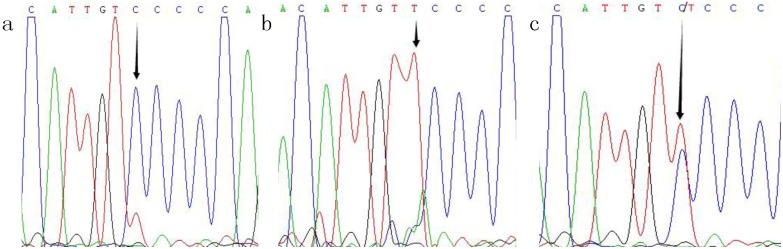
Sequencing map of the genotype for the IL-4−589C/T polymorphism. Arrow in parts a−c indicates CC, TT and C/T genotypes, respectively.

**Figure 3:**
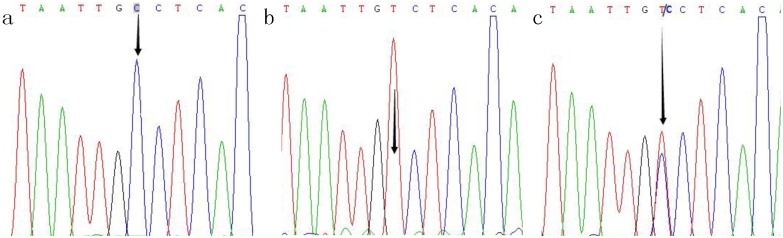
Sequencing map of the genotype for the IL-4−33C/T polymorphisms. Arrow in parts a−c indicates CC, TT and C/T genotypes, respectively.

By using the standard phenol-chloroform method, genomic DNA was extracted from 2 mL peripheral white blood cells. The −589C/T and −33C/T SNPs in *IL-4* were detected by polymerase chain reaction-restriction fragment length polymorphism (PCR-RFLP). Primers and probes were designed by Sangon Biotech Company (Shanghai, China). The primer sequences, annealing temperature, length of the PCR products, and corresponding restriction enzyme used for genotyping are presented in Table S1. After amplification, the PCR product was digested by restriction enzyme, visualized on a 2.5% agarose gel, and stained with ethidium bromide (Promega Corporation, Madison, WI, USA) for genotyping (Figure 1). The genotyping was performed for all participants under blinded conditions. For quality control, 50% of samples were repeated and direct sequencing was also performed by Sangon Biotech Company using 10% randomly selected samples (Figures 2 and 3); a 100% concordance rate was achieved.

### Statistical analysis

The statistical analyses were performed using the statistical software package SPSS 16.0 (SPSS Inc., Chicago, IL, USA). The Hardy-Weinberg equilibrium (HWE) was firstly tested with a goodness-of-fit χ^2^ test. General characteristics among the four groups were determined via Analysis of Variance (ANOVA) and the allele frequency and genotype distribution of *IL-4* gene polymorphisms were compared using the χ^2^ test and Fisher’s exact test, when appropriate. Binary logistic regression was used to calculate the odds ratios (ORs) and 95% confidence intervals (CIs) after age and gender status adjustments. Globally, HCC rates are more than twice as high in males as in females [25] and, therefore, gender may be a possible confounder. Thus, a stratification of the study population into males and females was further performed to evaluate the association within each stratum. Considering the possible linkage disequilibrium between the polymorphisms, Shi’s standardized coefficient D’ was used for quantification [26]; using the Phase program [27], [28], haplotypes and their frequencies were also estimated based on a Bayesian algorithm. All significance tests were two-sided, and *P*<0.05 was considered a statistically significant difference.

## Results

### Characteristics of the study participants

Table 1 summarizes the basic characteristics of the 170 healthy controls and the 129 CHB, 62 LC, and 154 HCC patients. Briefly, the LC and HCC patients were, on average, 10 years older than CHB patients and healthy controls, with the difference being statistically significant (both *P*<0.001). As for gender distribution, there were no significant differences between the case and control groups (*P* = 0.488, *P* = 0.618, and *P* = 0.751, respectively).

**Table 1:** Basic characteristic of the study population.

Variable	HealthyControls(n = 170)	CHB patients(n = 129)	*P*-value	LC patients(n = 62)	*P*-value	HCC patients(n = 154)	*P*-value
Age(year, mean±SD)	37.68±11.76	37.67±7.78	0.994	47.38±9.64	<0.001	49.19±11.33	<0.001
Gender, N(%)							
Male	147(86.5)	115(89.1)	0.488	52(83.9)	0.618	135(87.7)	0.751
Female	23(13.5)	14(10.2)		10(16.1)		19(12.3)	

### Genotype polymorphisms and the risk of HBV-related diseases

The genotype and allele frequencies of the −589C/T and −33C/T *IL-4* gene polymorphisms for patients with HBV-related diseases and healthy controls are shown in Table 2. According to the HWE test, the genotype distribution of the two SNPs in the patient groups and healthy controls were all agreed with HWE (*P*>0.05 for all). However, no significant differences between the genotype and allele frequencies of the −589C/T and −33C/T *IL-4* gene polymorphisms and CHB risk were observed. Binary logistic regression analyses adjusted for age and gender also failed to reveal any significant difference between them. Further, when considering LC and HCC patients, similar non-significant results were also found.

**Table 2:** Genotype and allele frequencies of −589C/T and −33C/T polymorphisms between HBV-related patients and healthy controls.

Polymorphisms	Healthycontrols,N = 170(%)	CHBpatientsN = 129(%)	LCpatients,N = 62(%)	HCCpatients,N = 154(%)	CHB patientsvs. Healthycontrols	LC patients vs.Healthy controls	HCC patientsvs. Healthycontrols
					OR (95%CI)^a^		
−589C/T							
TT	115(67.6)	77(59.7)	37(59.7)	111(72.1)	1.00	1.00	1.00
CT	51(30.0)	43(33.3)	22(35.5)	39(25.3)	1.28(0.78–2.12)	1.58(0.80–3.10)	0.90(0.52–1.56)
CC	4(2.4)	9(7.0)	3(4.8)	4(2.6)	3.35(0.99–11.28)	2.10(0.39–11.34)	1.13(0.26–5.06)
Dominant model^b^	55(32.4)	52(40.3)	25(40.3)	43(27.9)	1.44(0.89–2.33)	1.56(0.81–2.99)	0.921(0.54–1.56)
Recessive model^c^	166(97.6)	120(93.0)	59(95.2)	150(97.4)	3.08(0.93–10.25)	1.92(0.87–7.36)	1.17(0.27–5.13)
T allele	281(82.6)	197(76.4)	96(77.4)	261(84.7)	1.00	1.00	1.00
C allele	59(17.4)	61(23.6)	28(22.6)	47(15.3)	1.49(0.99–2.23)	1.51(0.87–2.61)	0.95(0.60–1.51)
P-_HWE_	0.550	0.383	0.907	0.796			
−33C/T							
TT	113(66.5)	77(59.7)	36(58.1)	97(63.0)	1.00	1.00	1.00
CT	52(30.6)	42(32.6)	22(35.4)	49(31.9)	1.21(0.73–2.00)	1.59(0.81–3.13)	1.29(0.76–2.20)
CC	5(2.9)	10(7.8)	4(6.5)	8(5.2)	2.92(0.96–8.92)	2.33(0.52–10.56)	2.24(0.65–7.74)
Dominant model^b^	57(33.5)	52(40.3)	26(41.9)	57(47.1)	1.36(0.84–2.20)	1.67(0.87–3.19)	1.38(0.83–2.30)
Recessive model^c^	165(97.1)	119(92.2)	58(93.5)	146(94.8)	2.74(0.91–8.25)	1.98(0.25–8.74)	2.06(0.60–7.01)
T allele	278(81.8)	196(76.0)	94(75.8)	263(78.9)	1.00	1.00	1.00
C allele	62(18.2)	62(24.0)	30(24.2)	65(21.1)	1.43(0.96–2.13)	1.56(0.91–2.66)	1.38(0.90–2.12)
P-_HWE_	0.737	0.219	0.797	0.581			

a Adjusted by age and gender;

b Dominant model: CT+CC versus TT;

c Recessive model: CC versus TT+CT.

Nevertheless, when subgroup analyses between males and females were conducted, significant differences were found in males in the CC genotype in co-dominant genetic model CC versus TT and recessive genetic model CC versus TT+CT. Both 589C/T CC and −33C/T CC genotypes in males were associated with a significantly increased risk of CHB compared with the TT genotype (OR: 4.193, 95% CI: 1.094–16.071, and *P* = 0.037; and OR: 3.438, 95% CI: 1.032–11.458, and *P* = 0.044); similar situation was also found in the recessive model (OR: 4.09, 95% CI: 1.08–15.49, and *P* = 0.038; and OR: 3.43, 95% CI: 1.04–11.28, and *P* = 0.042) (Table 3). On the other hand, significant differences between case and control groups were not observed for any genotype in females (Table S2).

**Table 3:** Genotype and allele frequencies of −589C/T and −33C/T polymorphisms between HBV-related patients and healthy controls in males.

Polymorphisms	Healthycontrols,N = 147(%)	CHBpatientsN = 115(%)	LCpatients,N = 52(%)	HCCpatients,N = 135(%)	CHB patients vs.Healthy controls,OR(95%CI)^a^	LC patients vs.Healthycontrols, OR(95%CI)^a^	HCC patientsvs. Healthycontrols, OR(95%CI)^a^
−589C/T							
Genotypes							
TT	100(68.0)	72(62.6)	31(59.6)	95(70.4)	1.00	1.00	1.00
CT	44(30.0)	34(29.6)	19(36.5)	36(26.6)	1.08(0.63–1.86)	1.92(0.92–4.01)	1.01(0.57–1.81)
CC	3(2.0)	9(7.8)	2(3.9)	4(3.0)	**4.19(1.09–16.07)** *	2.83(0.42–18.88)	1.59(0.32–7.98)
Dominant modelb	47(32.0)	43(37.4)	21(40.4)	40(29.6)	1.28(0.76–2.15)	1.98(0.99–4.08)	1.05(0.60–1.85)
Recessive modelc	144(98.0)	106(92.2)	50(96.1)	131(97.0)	**4.09(1.08–15.49)** *	2.23(0.35–14.43)	1.58(0.32–7.88)
T allele	244(83.0)	178(77.4)	81(77.9)	226(83.7)	1.00	1.00	1.00
C allele	50(17.0)	52(22.6)	23(22.1)	44(16.3)	1.43(0.93–2.06)	1.76(0.97–3.18)	1.085(0.67–1.77)
−33C/T							
Genotypes							
TT	98(66.7)	72(62.6)	30(57.7)	84(62.2)	1.00	1.00	1.00
CT	45(30.6)	33(28.7)	19(36.5)	43(31.9)	1.01(0.58–1.74)	1.84(0.89–3.82)	1.37(0.78–2.43)
CC	4(2.7)	10(8.7)	3(5.7)	8(5.9)	**3.44(1.03–11.46)** *	2.76(0.51–14.96)	2.96(0.78–11.22)
Dominant modelb	49(33.3)	43(37.4)	22(42.3)	51(37.8)	1.20(0.72–2.01)	1.92(0.95–3.89)	1.51(0.87–2.61)
Recessive modelc	143(97.3)	105(91.3)	49(94.2)	127(94.1)	**3.43(1.04–11.28)** *	2.22(0.43–11.54)	2.65(0.71–9.90)
T allele	241(82.0)	177(77.0)	79(76.0)	211(78.1)	1.00	1.00	1.00
C allele	53(18.0)	53(23.0)	24(24.0)	59(21.9)	1.37(0.89–2.10)	1.73(0.97–3.07)	1.52(0.96–2.41)

a Adjusted by age and gender;

b Dominant model: CT+CC versus TT;

c Recessive model: CC versus TT+CT;

*p<0.05.

Considering that the *IL-4* genetic background may be distinct between different populations, the genotype and allele frequencies of the two SNPs in our control group were further compared with those in different races from the Haplotype Map (HapMap) project (http://www.ncbi.nlm.nih. gov/snp/) as well as in previous reports [29]. The data shown in Table 4 suggests that the distribution of the two SNPs in the present study is significantly different from that in JPT (Japanese in Tokyo) and CEU (Utah residents with northern and western European ancestry) populations. For the 589C/T polymorphism, the frequencies of genotype TT and allele T in JPT and CEU populations are significantly lower, and the rate of CC genotype and C allele are significantly higher. With respect to the −33C/T polymorphism, significant genotype differences between the present study and YRI (Yoruba in Ibadan) were also found. Further, there were significantly lower detection rates of the TT genotype and T allele and higher detection rates of the CC genotype and C allele in the JPT, CEU, and YRI populations when compared with our data.

**Table 4:** Comparison of genotype and allele frequencies in the healthy control subjects of our study and that from the HapMap project.

Polymorphisms	Samples, N	Genotype frequency, n(%)				Alleles frequency, n(%)		
		TT	CT	CC	P values	T	C	P values
−589C/T								
Present study	170	115(67.6)	51(30.0)	4(2.4)		281(82.6)	59(17.4)	
HCB*	86	50(58.1)	34(39.5)	2(2.3)	0.307	134(77.9)	38(22.1)	0.196
JPT*	170	90(52.9)	66(38.8)	14(8.2)	0.005	246(72.4)	94(27.6)	0.001
CEU*	226	4(1.8)	54(23.9)	168(74.3)	0.000	62(13.7)	390(86.3)	0.000
YRI*	226	140(61.9)	76(33.6)	10(4.4)	0.356	356(78.8)	96(21.2)	0.172
Chinese†	151	92(60.9)	50(33.1)	9(6.0)	0.185	234(77.5)	68(22.5)	0.101
−33C/T								
Present study	170	113(66.5)	52(30.6)	5(2.9)		278(81.8)	62(18.2)	
HCB*	86	50(58.1)	34(39.5)	2(2.3)	0.357	134(77.9)	38(22.1)	0.298
JPT*	172	90(52.3)	68(39.5)	14(8.1)	0.011	248(72.1)	96(27.9)	0.003
CEU*	226	4(1.8)	54(23.9)	168(74.3)	0.000	62(13.7)	390(86.3)	0.000
YRI*	226	44(19.5)	126(55.8)	56(24.8)	0.000	214(47.3)	238(52.7)	0.000
HCH†	159	104(65.4)	51(32.1)	4(2.5)	0.939	259(81.4)	59(18.6)	0.916

*Data from HapMap Project;

† Data from previous reports; HCB, Han Chinese in Beijing, China; JPT, Japanese in Tokyo, Japan; CEU, Utah residents with northern and western European ancestry; YRI, Yoruba in Ibadan, Nigeria; HCH, Han Chinese in Hunan province, China.

### Estimated haplotype frequencies and the risk of HBV-related diseases

Haplotype analyses were performed for all patient groups and healthy controls using the SHEsis software, and the four possible haplotype frequencies of the two SNPs are shown in Table 5. According to the results, the T^−589^ T^−33^ haplotype is the most common haplotype and represents >50% in all groups, whereas the C^−589^ C^−33^ haplotype accounts for 14.9% to 23.3% among the four groups and represents the second highest haplotype. After haplotype analyses, however, no significant differences in these haplotype frequencies were found in any group. Finally, C^−589^ T^−33^ and T^−589^ C^−33^ are rare haplotypes that account for less than 2% in the control subjects and therefore further analyses were not conducted for these.

**Table 5:** Frequencies of the haplotypes formed by −589C/T and −33C/T polymorphisms in HBV-related patients and healthy controls.

Haplotype	Healthycontrol(%)	CHBPatients(%)	OR(95%CI)	p	LCPatients(%)	OR(95%CI)	p	HCCPatients(%)	OR(95%CI)	p
CC	17.1	23.3	1.47(0.98–2.20)	0.061	16.5	0.96(0.55–1.66)	0.878	14.9	0.85(0.56–1.30)	0.459
CT	0.3	0.4	-	-	6.1	-	-	0.3	-	-
TC	1.2	0.8	-	-	7.7	-	-	6.2	-	-
TT	81.5	75.6	0.68(0.45–1.02)	0.061	69.7	0.52(0.33–0.84)	0.064	78.6	0.83(0.57–1.23)	0.356

## Discussion

China, a region with 94 million HBsAg carriers, accounts for 50% of the total HCC cases worldwide [30], [31]. The predominant mode of HBV transmission in this hyperendemic region is vertical transmission from infected mothers during or shortly after birth [32]. However, only 5% to 10% of perinatally-infected infants become persistent carriers as adults, despite the approximately 90% risk; further, from these persistent infectors, 10% to 30% will progress to LC and HCC [33]. Such highly variable disease outcomes cannot be fully explained by differences in immunological, viral, or environmental factors. Thus, differences in host genetic factors may play essential roles in these processes.

IL-4, a typical pleiotropic Th2 cytokine [9], is best known for defining the Th2 phenotype of lymphocytes, as well as being involved in regulating cell proliferation, apoptosis, and expression of numerous genes in lymphocytes, macrophages, and fibroblasts, among others [34]–[39]. Functional variations in this gene may contribute to viral clearance as well as HBV-associated HCC in high-risk Chinese patients. However, in the present study, we did not observe any association of *IL-4* genotypes, alleles, and haplotypes with the overall CHB, LC, and HCC patients. Nevertheless, in subgroup analysis, CC genotypes of the *IL-4* −589C/T and −33C/T polymorphisms were observed to be significantly associated with CHB in males as compared with the TT genotypes and TT+CT genotypes. In females, a similar trend towards an increased risk effect of CC genotypes in CHB was also observed, but the difference was not significant. These results indicate that *IL-4* gene polymorphisms may be a risk factor for CHB in the Chinese male population only.

Data from previous epidemiological studies was consistent with our results. In a meta-analysis conducted by Zheng et al. [20] exploring the *IL-4* 590C/T polymorphism and its susceptibility to liver disease (including HBV infection, HCV infection, liver cirrhosis, etc.), significant associations between the *IL-4* 590T polymorphism and increased risk of liver diseases was found in Caucasian populations, but not in Asian populations. Another meta-analysis investigating the association of *IL-4* polymorphisms with response to HBV vaccine and susceptibility to HBV infection, also found no evidence indicating a correlation between *IL-4* polymorphisms (rs2243250 C>T) and susceptibility to HBV infection [21]. It is noted that all “Asian population” studies included in the two meta-analyses mentioned above were conducted in China [40]–[45].

It is noteworthy that, in the present study, the significant association between CHB in males was found in the CC genotypes of −589C/T and −33C/T polymorphisms, as opposed to the T allele in the Caucasian populations studied in the meta-analysis of Zheng et al. [20]. The fact that the same polymorphism site has distinct genotype frequencies which play different roles in the same disease may be principally explained by differences in the *IL-4* genetic background among ethnicities; results from the comparison of genotype and allele frequencies in the healthy control subjects in the present study and those from the HapMap project provide evidence to this effect. In the current study, the *IL-4* −590T and −33T allele frequencies among healthy controls were 82.6% and 81.8%, respectively, which were significantly higher than those observed in healthy Caucasians (both were 13.7%), while the C allele frequencies accounted for 17.4% in −590C/T mutations and 18.2% in −33C/T mutations, which were significantly lower than in Caucasians (both were 86.3%). Thus, the T allele may be the major allele in −589C/T and −33C/T SNPs in the Chinese population, and the C allele is the variant responsible for various diseases, while the opposite is true for Caucasians.

With respect to HCC, only two studies were found regarding the *IL-4* −590 C/T polymorphism and HCC risk in a high-risk Chinese population [46] and in low-risk non-Asians in the USA [22], both conducted by Ognjanovic et al. [22], [46]. Contrary to our results, the authors noted a substantial decrease in HCC risk associated with the *IL-4* −590 CC genotype in Guangxi, China, and a non-significant result in Los Angeles non-Asians. However, in the present study, no association was found for either *IL-4* −590C/T or −33C/T polymorphisms and HCC risk in the Chinese population. Drinking status may be a possible factor in the disparate associations between our study and theirs. According to their results [46], the *IL-4* −590 CC genotype was not associated with a lower risk of HCC when initially adjusted for age, gender, and ethnicity (CC vs. CT/TT, OR: 0.46, 95% CI: 0.16–1.31); however, after further adjustment for HBsAg seropositivity and the number of alcoholic drinks per day (0, <3, or >3), the CC genotype was found to be negatively associated with HCC risk (CC vs. CT/TT, OR: 0.05, 95% CI: 0.01–0.40); in contrast, individuals included in our study were all HBsAg seropositive. In addition, the difference in study design and sample size may also be possible confounders.

For *IL-4* polymorphism haplotypes, previous experimental data has demonstrated some to be associated with respiratory syncytial virus [47], multiple sclerosis [48], oral cancer [49], and systemic lupus erythematosus [50], suggesting that certain important polymorphisms could affect the regulation of this cytokine. In addition, the T allele of the *IL-4* −590C/T polymorphism has been reported to be in linkage disequilibrium with −33T [51], and was associated with an increased expression of IL-4 [52]. However, we did not find an association between any of the estimated haplotype frequencies and the risk of HBV-related diseases, indicating that the *IL-4* gene haplotypes may not play any facilitative role in the development of HCC.

In conclusion, we could not detect an effect of genetic variants between *IL-4* and HBV-related HCC in a Chinese population. Interestingly, in subgroup analysis, the CC genotype of *IL-4* −589C/T and −33C/T polymorphisms were observed to be significantly correlated with the risk of CHB in the Chinese male population. However, considering the relatively small sample size of this study, more studies with a larger sample size are warranted in an attempt to confirm the observed association.

## Supporting Information

Table S1 **The sequences of forward and backward primers and restriction enzymes for genotyping IL-4 polymorphisms.**
(DOCX)

Table S2 **Genotype and allele frequencies of −589C/T and −33C/T polymorphisms between HBV-related patients and healthy controls in females.**
(DOCX)
