# Supplementary material for: Role of IL-4 Gene Polymorphisms in HBV-Related Hepatocellular Carcinoma in a Chinese Population
Source: PLoS One. 2014 Oct 8;9(10):e110061. doi: 10.1371/journal.pone.0110061 (PMC4190355; doi:10.1371/journal.pone.0110061)
Supplement: Table S1 — The sequences of forward and backward primers and restriction enzymes for genotyping IL-4 polymorphisms. (DOCX) [file pone.0110061.s001.docx]

Table S1. The sequences of forward and backward primers and restriction enzymes for genotyping IL-4 polymorphisms

| Polymorphisms | Primer sequence | Annealing temperature (℃) | | Restriction enzyme | PCR product (bp) |
| --- | --- | --- | --- | --- | --- |
| -589C/T | F:5’AACACCTAAACTTGGG AGGA3’ | 71 | PsyI(Tth111I) | | TT: 149 |
|  | R:5’CTGTCATGGAAAAGCTGATCT3’ |  |  | | CC: 107+42 |
|  |  |  |  | | CT: 149+107+42 |
| -33C/T | F:5’GCCCCAAGTGACTGACAATC3’ | 60 | Alw26I(BsmAI) | | CC: 182+129 |
|  | R:5′TCACCTTCTGCTCTGTGTGAGG3’ |  |  | | TT: 144+38+129 |
|  |  |  |  | | CT: 182+144+38+129 |
