# Supplementary material for: Role of IL-4 Gene Polymorphisms in HBV-Related Hepatocellular Carcinoma in a Chinese Population
Source: PLoS One. 2014 Oct 8;9(10):e110061. doi: 10.1371/journal.pone.0110061 (PMC4190355; doi:10.1371/journal.pone.0110061)
Supplement: Table S2 — Genotype and allele frequencies of −589C/T and −33C/T polymorphisms between HBV-related patients and healthy controls in females. (DOCX) [file pone.0110061.s002.docx]

Table S2. Genotype and allele frequencies of -589C/T and -33C/T polymorphisms between HBV-related patients and healthy controls in females.

| Polymorphisms | Healthy controls, N=23(%) | CHB patients  N=14(%) | LC patients, N=10(%) | HCC patients, N=19(%) | CHB patients vs. Healthy controls, OR (95%CI)^a^ | LC patients vs. Healthy controls, OR (95%CI)^a^ | HCC patients vs. Healthy controls, OR (95%CI)^a^ |
| --- | --- | --- | --- | --- | --- | --- | --- |
| -589C/T |  |  |  |  |  |  |  |
| Genotypes |  |  |  |  |  |  |  |
| TT | 15(65.2) | 5(35.7) | 6(60.0) | 16(84.2) | 1.00 | 1.00 | 1.00 |
| CT | 7(30.4) | 9(64.3) | 3(30.0) | 3(15.8) | 4.25(0.99-18.34) | 0.16(0.01-2.61) | 0.32(0.05-1.90) |
| CC | 1(4.4) | 0(0) | 1(10.0) | 0(0) | — | 0.26(0.003-21.49) | — |
| Dominant model^b^ | 8(34.8) | 9(64.3) | 4(40.0) | 3(15.8) | 3.78(0.90-15.96) | 0.17(0.01-2.61) | 0.27(0.05-1.60) |
| Recessive model^c^ | 22(95.6) | 14(100) | 9(90.0%) | 19(100) | — | 0.78(0.02-27.11) | — |
| T allele | 37(80.4) | 19(67.9) | 15(75.0) | 35(92.1) | 1.00 | 1.00 | 1.00 |
| C allele | 9(19.6) | 9(32.1) | 5(25.0) | 3(7.9) | 2.06(0.69-6.15) | 0.17(0.02-1.30) | 0.30(0.07-1.40) |
| -33C/T |  |  |  |  |  |  |  |
| Genotypes |  |  |  |  |  |  |  |
| TT | 15(65.2) | 5(35.7) | 6(60.0) | 13(68.4) | 1.00 | 1.00 | 1.00 |
| CT | 7(30.4) | 9(64.3) | 3(30.0) | 6(31.6) | 4.25(0.99-18.34） | 0.16(0.01-2.61） | 0.72(0.14-3.61） |
| CC | 1 (4.4) | 0(0) | 1(10.0) | 0(0) | — | 0.26(0.003-21.49） | — |
| Dominant model^b^ | 8(34.8) | 9(64.3) | 4(40.0) | 6(31.6) | 3.78(0.90-15.96) | 0.17(0.01-2.61) | 0.62(0.13-2.99) |
| Recessive model^c^ | 22(95.6) | 14(100) | 9(90.0%) | 19(100) | — | 0.78(0.02-27.11) | — |
| T allele | 37(80.4) | 19(67.9) | 15(75.0) | 32(84.2) | 1.00 | 1.00 | 1.00 |
| C allele | 9(19.6) | 9(32.1) | 5(25.0) | 6(15.8) | 2.06(0.69-6.15) | 0.39 (0.07-2.20) | 0.58(0.15-2.24) |

^a^ Adjusted by age and gender; ^b^ Dominant model: CT+CC; ^c^ Recessive model: TT+CT.
